# Controlling the Phase Behavior and Reflection of Main-Chain Cholesteric Oligomers Using a Smectic Monomer

**DOI:** 10.3390/ijms23063275

**Published:** 2022-03-18

**Authors:** Lansong Yue, Xiuyi Shi, Guofu Zhou, Laurens T. de Haan

**Affiliations:** 1 SCNU-TUE Joint Lab of Device Integrated Responsive Materials (DIRM), National Center for International Research on Green Optoelectronics, South China Normal University, Guangzhou 510006, China; 2019023206@m.scnu.edu.cn (L.Y.); xiuyi@m.scnu.edu.cn (X.S.); 2 Guangdong Provincial Key Laboratory of Optical Information Materials and Technology & Institute of Electronic Paper Displays, South China Academy of Advanced Optoelectronics, South China Normal University, Guangzhou 510006, China

**Keywords:** cholesteric reflection, stimuli-responsive, pre-transition effect, main-chain oligomers

## Abstract

Cholesteric liquid crystals (CLCs) are a significant class of temperature-responsive photonic materials that have the ability to selectively reflect light of a specific wavelength. However, the fabrication of main-chain CLC oligomers with dramatic reflection band variation upon varying the temperatures remains a challenge. Here, a feasible method for improving and controlling the responsiveness of main-chain cholesteric liquid crystal oligomers by the incorporation of a smectic monomer is reported. The smectic monomer strengthens the smectic character of the oligomers and enhances the magnitude of the change of the pitch as a function of temperature upon approaching the cholesteric–smectic phase transition temperature. The central wavelength of the reflection band can be easily modified by mixing in an additional chiral dopant. This promising method will open the door to the preparation of temperature-responsive photonic devices with excellent responsiveness.

## 1. Introduction

Temperature-responsive photonic materials are smart materials that are able to change their optical properties in response to temperature changes [1,2,3]. This property is of great use for various applications, including smart reflectors [4,5], anti-counterfeit devices [6], and optical devices [7,8]. Cholesteric liquid crystals (CLCs) are a major class of temperature-responsive photonic materials that have the ability to selectively reflect a specific wavelength of light due to their periodic helical molecular organization [9,10,11]. This wavelength is dependent on the helical pitch (the distance of one full director rotation) [4,12,13]. Thus, a change in pitch results in a change of the color of the CLC film, and there are many mechanisms through which this can be achieved, such as changes in the concentration of the chiral dopant [14,15,16], intensity of an electric field [17], thickness of the cross-linked film [18], thermal helical twisting power variation [19], cholesteric–smectic pre-transitional effect [20,21], phase separation [22,23,24] and thermal expansion [25].

Of particular interest is an effect that takes place in some cholesteric liquid crystals when the temperature is lowered toward the cholesteric–smectic phase transition, called the pre-transitional effect, which has been studied theoretically and experimentally [26,27,28,29]. Upon approaching the cholesteric–smectic transition point, a dramatic increase in pitch and a corresponding red shift of the reflection band are often observed [16,27,30,31]. This effect is understood as the gradual formation of short-range smectic order in the cholesteric helix [20,32,33,34], which leads to unwinding of the cholesteric helix and enlarges the pitch [35,36,37,38]. To obtain temperature-responsive photonic devices showing good stability and a large difference in reflection band position between low and high temperatures (the band-shift range) as a result of the pre-transition effect, the use of polymers is desired to stabilize the performance of the photonic materials [4,10,11,39,40,41,42]. A polymer-stabilized cholesteric liquid crystal in a cell containing a 5 wt% cross-linked network only showed a 150 nm reflection band shift as the temperature decreased from 45 °C to 15 °C, indicating a poor pre-transitional effect [43]. A temperature-responsive photonic coating based on cholesteric oligosiloxane liquid crystal with a 3 wt% cross-linked liquid crystal network only showed a 220 nm reflection band shift between 61 °C and 22 °C [26]. This shows that in many cases the mobility of the mesogens is largely lost by full or partial cross-linking of the materials, causing the suppression or loss of the pre-transitional effect [9,10,44]. It remains a challenge to properly control this effect in a polymeric temperature-responsive photonic system.

In our previous work, we prepared a photonic coating of main-chain CLC oligomers based on the thermally driven Michael addition between an acrylate and a primary amine, which showed variation of the reflection band over a range of several hundreds of nanometers [10]. In the current study, we designed and prepared novel main-chain CLC oligomers through the incorporation of smectic liquid crystal monomers into this previous design and studied their behavior. A drastic shift of the reflection band of the planarly aligned CLC oligomers upon cooling was observed with increasing the amount of smectic monomer, indicating an amplified cholesteric–smectic pre-transitional effect and improvement in the temperature-responsive properties. The band-shifting range of the planarly aligned CLC oligomers can be fine-tuned by changing the ratio of smectic and nematic monomer and mixing with an additional chiral dopant. To show this, a series of main-chain CLC oligomers with a cholesteric–smectic phase transition that contained different concentrations of smectic monomer and chiral dopant was synthesized. This feasible method for controlling the pre-transitional effect of main-chain CLC oligomers has great potential to be utilized in temperature-responsive photonic devices such as IR reflectors, anti-counterfeiting labels, smart wearable optical devices, and so on.

## 2. Results and Discussion

### 2.1. Synthesis of the Main-Chain CLC Oligomer

**Figure 1:**
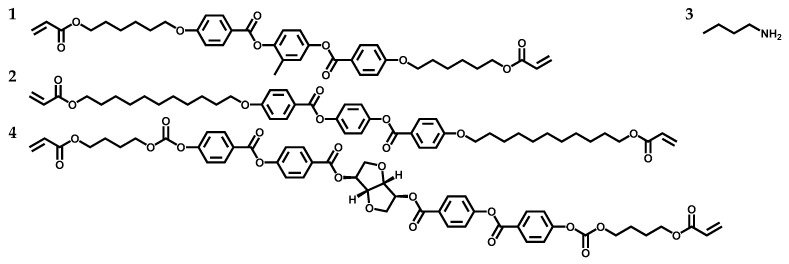
Monomer composition of the main-chain CLC oligomer used.

**Figure 2:**
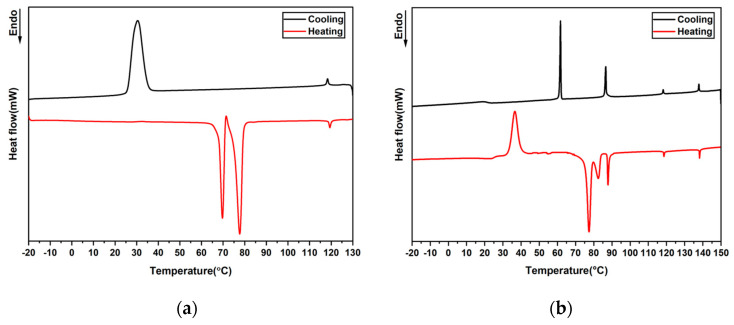
(**a**) DSC curves of pure Monomer 1. (**b**) DSC curves of pure Monomer 2.

The synthesis of the main-chain CLC oligomers was carried out using a previously described procedure, by mixing achiral LC Monomer 1, achiral LC Monomer 2, right-handed chiral LC Monomer 4, and butylamine 3 [10,45,46,47,48]. The chemical structures of all materials are shown in Figure 1. During the polymerization, a repeated Michael addition reaction takes place. Control of the degree of polymerization (DP) of the oligomer is achieved by adjusting the ratio between acrylate groups and amine groups. Monomer 1 is a nematic diacrylate liquid crystal monomer (see the differential scanning calorimetry (DSC) curves in Figure 2a), and Monomer 2 is a diacrylate liquid crystal monomer that can exhibit smectic and nematic liquid crystal phases (see the DSC curves in Figure 2b and polarizing optical microscopy (POM) images in Figure S1). Butylamine 3 acts as a chain extender for the Michael addition reaction. Monomer 4 is a chiral dopant for the formation of the cholesteric liquid crystal phase. The mixture was dissolved in tetrahydrofuran (THF) and stirred at 25 °C for 24 h to ensure the formation of secondary amines. Then, the reaction was carried out at 100 °C for another 18 h to form tertiary amines and complete the oligomerization. The vial was placed in a vacuum oven at 50 °C overnight to remove the remaining THF. The material was viscous and stable at room temperature for months, similar to the material reported in our previous research [10]. By varying the ratio of LC Monomer 2 to Monomer 1, specific compositions were obtained. The molar ratios of LC Monomer 1 to Monomer 2 for each sample are listed in Table 1. The concentration of the chiral dopant was always kept at 5 mol%.

To determine the structure and average degree of polymerization of the main-chain CLC oligomers with different concentrations of Monomer 2, all samples were characterized by ^1^H NMR (Figure S2). By comparing the values of the peaks corresponding to the three diacrylate end groups and peaks corresponding to the aromatic protons in the aromatic groups, the average DP of all the oligomers was calculated and is shown in Table 1. The DP was kept relatively small on purpose, as higher DP values lead to red shift of the reflection band, outside of the measurement range of the equipment used to measure them in later experiments.

### 2.2. CLC Oligomers Phase Behavior

**Figure 3:**
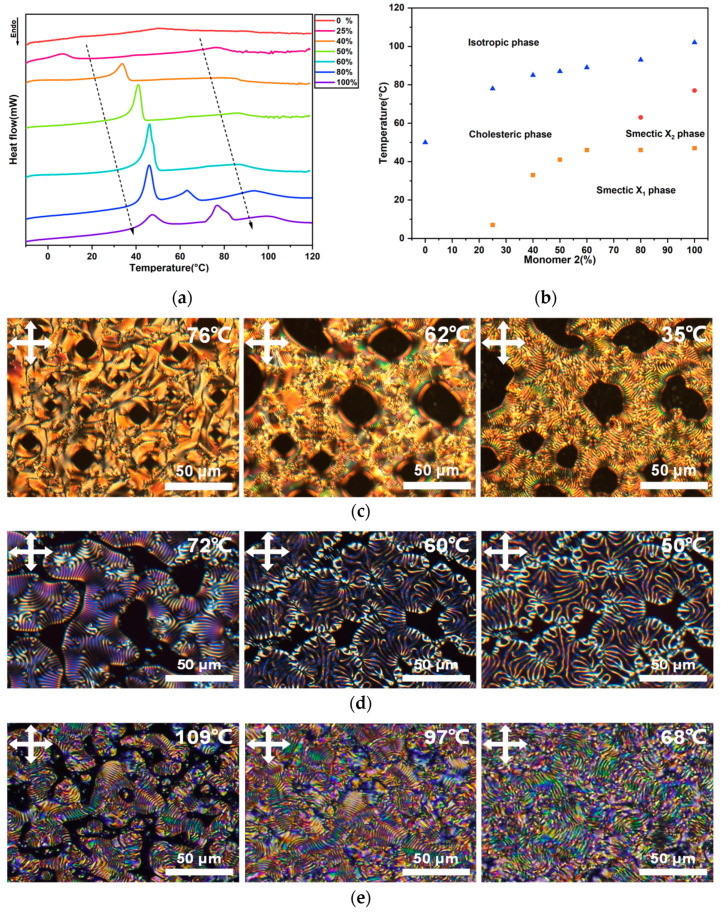
(**a**) DSC thermograms of the CLC oligomers during the cooling cycle at a cooling rate of 5 °C/min. (**b**) Phase diagram of CLC oligomers containing different concentrations of Monomer 2. (**c**) POM images of CLC oligomers with 40%, (**d**) 80%, and (**e**) 100% Monomer 2 at various temperatures under cooling.

The phase behaviors of the CLC oligomers with different compositions were studied. DSC was measured between −10 °C and 120 °C to find the phase transition temperatures (Figure 3a,b). After aligning the oligomers by shear force between two flat glass substrates, the samples with obvious defect structure were selected so that the characteristic textures of the CLC oligomers with different concentrations of Monomer 2 could be determined using POM under slow cooling (Figure 3c–e).

When the oligomer contained 0% Monomer 2, one exothermic peak was found from the DSC curve, corresponding to the cholesteric–isotropic phase transition, which has been systematically discussed in our previous research [10]. As the Monomer 2 content was increased to 25%, DSC curves revealed a marked increase in the temperature of the cholesteric–isotropic phase transition point, from 50.8 °C to 78.2 °C, and a noted exothermic peak representing a cholesteric–smectic phase transition. The oligomer displayed a cholesteric phase that showed representative oily streak textures under POM above room temperature (Figure S3a). With 40% Monomer 2, the DSC diagram demonstrated phase transitions at further increased temperatures, with the isotropic transition point at 86 °C and the cholesteric–smectic transition point at 33 °C. The oligomer exhibited cholesteric focal-conic textures first at around 76 °C, then cholesteric fingerprint textures at 62 °C when cooled down from the isotropic phase, as shown in Figure 3c. The dark areas were examined by conoscopy to verify if there were possible homeotropic alignment. However, no proof of homeotropic alignment was found. As such, it is not clear what these areas represent. Approaching 33 °C, the distances between adjacent fingerprint texture lines gradually increased, corresponding to a gradual generation of short-range smectic order. At 50% and 60% Monomer 2, two similar exothermic phase transition peaks were observed, as well as obvious transformation of typical liquid crystal textures, which were similar to the behavior of oligomers containing 40% Monomer 2 (Figure S3b).

However, the oligomers prepared from 80% Monomer 2 exhibited surprisingly different peaks. We noted that one remarkable peak was detected around 65 °C between the cholesteric–smectic transition peak at 45 °C and the cholesteric–isotropic transition peak at 93 °C in the DSC curve, which indicates the presence of another mesophase. When monitored by POM, a significant change in texture of the mesophase was observed as the temperature was decreased (Figure 3d). At 72 °C, the representative fingerprint textures with a narrow distance of two adjacent stripes revealed a cholesteric phase. When cooling, changes in the order of fingerprint textures were noted, showing a gradual increase in the width between two adjacent stripes. The changes may correspond to the emergence of a new smectic mesophase (60 °C), which may be similar to textures reported before [49]. On further cooling to 50 °C, the width kept increasing, which indicated the continuous formation of smectic order, corresponding to the smectic phase in a temperature range between 45 °C and 65 °C. With a further increase in the concentration of Monomer 2 to 100%, the oligomer underwent a similar phase transition sequence that exhibited three phase transition peaks when detected by DSC and representative fingerprint textures when characterized under POM. DSC curves revealed a cholesteric–isotropic phase transition point at 100 °C, a subsequent cholesteric–smectic phase transition temperature at 76 °C, and a phase transition between the two smectic phases at 47 °C. Under POM, the oligomer was found to exhibit fingerprint textures with short helical pitches at 109 °C and 97 °C, corresponding to a cholesteric phase. However, the cholesteric–smectic phase transition observed upon cooling seems to accompany some broken focal-conic textures at 68 °C, which led to the formation of slightly longer width between two adjacent stripe lines in the fingerprint textures and a disordered structure in the cholesteric phase (Figure 3e), also suggesting that the helical pitch became longer.

From the DSC and POM analysis, it can be seen that the temperatures of both the cholesteric–smectic transition peak and the cholesteric–isotropic transition peak elevated gradually with the increase in smectic Monomer 2 concentration (Figure 3b). It also became clear that the increasing concentration of smectic monomers in the main-chain structure caused the formation of more smectic clusters in the cholesteric order as the temperature approached the cholesteric–smectic phase transition temperature. It is speculated that the increasing number of short-range smectic structures leads to unwinding of the cholesteric helix, leading to a pre-transitional effect in the cholesteric liquid crystals [37]. With the size of the smectic clusters growing, the pitch rapidly changes, thus presenting a strong unwinding of the helical structure before the transition from cholesteric to smectic phase. Therefore, we assume that the short-range temporary smectic ordering, which is enhanced by incorporating the smectic monomer into the main-chain oligomer, resulted in changes of the material’s alignment to unperfect planar alignment and explained the dramatic changes in textures as the pre-transitional point was approached.

**Figure 4:**
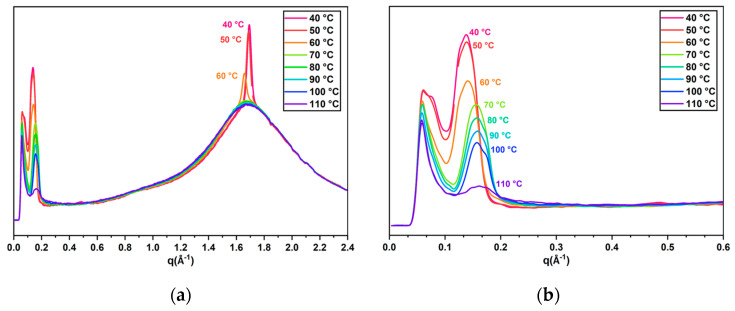
(**a**) XRD of CLC oligomers with 25% Monomer 2 at different temperatures under cooling. (**b**) Amplified peaks at q = 0.14 Å^−1^ for different temperatures, which indicate the existence of smectic phase order.

To further confirm the phase behavior caused by the smectic monomer, XRD measurement of the oligomer with 25% Monomer 2 was utilized to accomplish the mesophase identification (Figure 4a,b). The measurement showed a diffraction peak at different temperatures located at around 0.14 Å^−1^, which was an obvious indication of the smectic order due to its ordered structural arrangement. The peaks at around 1.7 Å^−1^ represent the side-by-side intermolecular distance, which can be detected in both the nematic and smectic phases. At higher temperatures, the peaks were diffuse with lower intensity. As the temperature decreased, the molecular orientation became more ordered, which led to the diffraction peak becoming sharper and more pronounced. The appearance of the smectic structure through the cholesteric phase made the peaks intensity increase with decreasing temperature, and an especially dramatic shift was observed when cooled down from 60 °C to 50 °C in both the wide- and small-angle section of the XRD spectra, indicating an obvious and gradual cholesteric-smectic pre-transitional effect of the cholesteric liquid crystal oligomers. In comparison to the XRD results, the POM and DSC data show an isotropic to cholesteric transition at ~80 °C and a cholesteric-to-smectic transition below room temperature. The temperature range is not consistent precisely with the temperature range found in the XRD data, indicating the wide range of the pre-transitional effect starting at 60 °C. While the increase in the intensity of the small-angle peak can be explained by an increase in smectic domains, the sharpening of the peak at 1.7 Å^−1^ is not so easily explained, as it should not be strongly affected by the presence of such domains. While it is possible that the sharpening of this peak is caused by the presence of exotic smectic phases in the system, there is currently not enough evidence to draw such conclusions. The results support that the pre-transitional effect is present and enhanced by the incorporation of Monomer 2.

### 2.3. Temperature Response of the Reflection Band of the Oligomers

**Figure 5:**
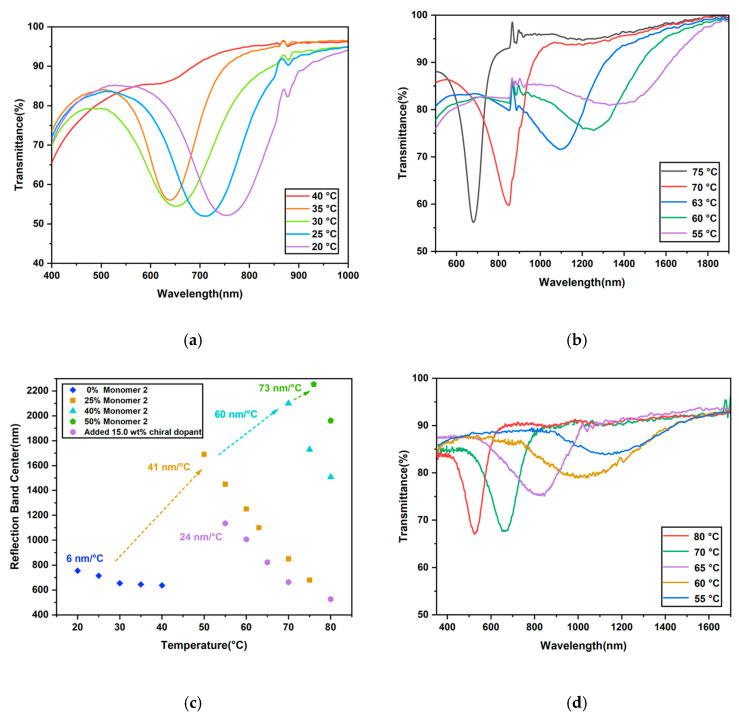
(**a**) Transmittance spectra of the oligomer with no Monomer 2 upon cooling. (**b**) Transmittance spectra of oligomer with 25% Monomer 2. (**c**) The central reflection wavelength of oligomers with different concentrations of Monomer 2 as a function of temperature upon cooling. In addition, the data for the oligomer with 50% Monomer 2 and an additional 15% chiral dopant is shown. (**d**) Transmittance spectra of the oligomer with 50% Monomer 2 after mixing with additional 15.0 wt% chiral dopant upon cooling.

Ultraviolet–visible–IR (UV–vis–IR) spectroscopy was used to study the temperature-responsive properties of the oligomers with various compositions. The most well-aligned samples, based on their transparency and POM images, were chosen for these experiments (Figure S4). By analyzing the transmittance spectra of the film at different temperatures, we compared the temperature response properties of the oligomers with different amounts of Monomer 2. The spectrum of the film with no Monomer 2 only showed a reflection band shift from 644 nm to 755 nm upon cooling from 40 °C to 20 °C, for a total of 111 nm red shift (Figure 5a). No reflection band could be measured above 40 °C. In our previous research, for the cholesteric liquid crystal oligomer with no Monomer 2 and containing less chiral dopant (4.3 mol%), when cooled down from 75 °C to 40 °C, the reflection band underwent a 175 nm red shift from 700 nm to 875 nm [10]. Upon further cooling from 40 °C to 20 °C, 225 nm red shift from 875 nm to 1100 nm was obtained. Although the position of the reflection band shifted, the steepness between 75 °C and 20 °C was only 7.27 nm/°C. In comparison, the spectrum of the oligomer with 25% Monomer 2 showed a 1010 nm band shift from 680 nm to 1690 nm between 75 °C and 50 °C, which showed a broader band-shift range (Figure 5b). Oligomers with a larger amount of Monomer 2 were also investigated. The central wavelengths of the reflection bands of different oligomers were plotted against temperature (Figure 5c).

For the oligomer with 40% Monomer 2, the reflection band underwent a 593 nm red shift upon cooling from 80 °C to 70 °C (Figure S5a). No further peaks were observed below 70 °C; one of the possible reasons for this is that the central wavelength already exceeded the measurement range of the instrument at 2500 nm. As the POM images of this film displayed fingerprint textures at 62 °C, another possibility is that the alignment of the helices changed from planar to homeotropic, which could account for the inability to reflect light with a specific wavelength. When 50% Monomer 2 was added, a 293 nm band shift from 1961 nm to 2254 nm was observed upon cooling from 80 °C to 75 °C (Figure S5b). Comparing this to the DSC curves and POM images, the quick increase in pitch may be caused by the increase in smectic order in the cholesteric phase when the temperature approaches 68 °C, resulting in a dramatic red shift of only 5 °C. Due to the limitation of the measurement range, it is not possible to measure the reflection band in the further cooling process.

For the oligomers containing 60%, 80%, and 100% Monomer 2, the spectroscopy data did not show any reflection band in the specific temperature measurement range, which also corresponded to the POM images. Larger amounts of smectic monomer took up the main part of the main-chain oligomer, which led to the formation of a large degree of smectic order upon cooling. This smectic order led to homeotropic alignment and that could explain the lack of a reflection band. Over the same temperature range, the oligomer with a high content of smectic monomer tended to present larger changes in the reflection band, which means higher variation and sensitivity per °C.

Repeat POM and UV–vis experiments using new batches of the oligomer with 25%, 40%, and 50% smectic monomer were performed to investigate the reproducibility of the experiments. The transmittance spectrum of the oligomer with 25% smectic monomer demonstrated an obvious reflection band, and a 1479 nm reflection band shift upon cooling from 75 °C to 40 °C. The oligomer with 40% Monomer 2 showed a 635 nm red shift upon cooling from 75 °C to 65 °C. The steepness between 75 °C to 65 °C was 63 nm/°C, similar to the previous results. For the oligomer containing 50% Monomer 2 similar results were obtained and the reflection band displayed a 397 nm red shift in only 5 °C. This data is summarized in Figure S6a. The temperature-dependent reflection wavelength centers of the aligned samples made from the new batches indicate good reproducibility when compared to the original data. It needs to be pointed out that the reflection band of the new samples had shifted a little bit compared to the previous samples, but due to the large effect of the smectic monomer on the reflective behavior of the material, this does not significantly affect the conclusions that can be drawn from the data.

The reflection wavelength is inversely proportional to the HTP (λ^−1^ ~ HTP). Comparison of the inverse reflection wavelength of the oligomers at different temperatures was performed to study the influence of temperature on HTP. As shown in Figure S7a, the values of the inverse central reflection wavelength of the oligomers with different concentrations of Monomer 2 all decreased upon cooling, indicating the proportional decrease in HTP. Thus, pitch increased, and the corresponding red shift of the reflection band was observed. For the new batches of oligomers (Figure S7b) and the oligomers with additional chiral dopants (Figure S7c), a similar trend on the HTP was observed with decreasing temperature as well. Moreover, the Boltzman relation between the inverse reflection wavelength and temperature was studied by plotting ln(λ^−1^) as a function of T^−1^ to check the linearity (Figure S8). R^2^ (goodness of fit) of all the fitting curves demonstrated high linearity.

In our past research, the reflective film based on cross-linkable cholesteric liquid crystal oligomer only showed a 496 nm red shift from 701 nm to 1195 nm upon cooling from 74 °C to 16 °C. Here, by introducing 25% smectic monomer into the main-chain structure, a 1479 nm band shift from 979 nm to 2458 nm was achieved upon cooling from 75 °C to 40 °C. For the oligomer with a higher concentration of smectic monomer, the shift of the reflection band became larger and larger over the same temperature range. The results presented above strongly confirmed that the addition of Monomer 2 can improve the pre-transitional effect and the resulting temperature-responsive properties. By building the proper amount of smectic monomer into the main-chain oligomer, it is possible to enhance the sensitivity of the main-chain cholesteric oligomers to temperature changes. Because of the formation of smectic order in the cholesteric phase, a gradual increase in the helical pitch and a larger pre-transitional effect is observed. The more smectic monomer we added, the stronger the tendency of the molecules to form a local layer structure in the cholesteric phase, leading to an amplified pre-transitional effect and a larger shift of the reflection band. The increase in pitch due to the formation of smectic domains can also be seen as an increase in distance between the fingerprint structures in the POM images, which further supports this mechanism. The addition of the smectic monomer also leads to the reflection band range shifting to the end of the near-infrared region, which broadens the temperature response range, opening up some potential in the field of energy saving.

### 2.4. Fabrication of a Mixture Containing Main-Chain CLC Oligomer and Chiral Dopant

With the amount of the smectic monomer increasing, the center of the reflection band gradually shifted to the end of the near-infrared region. To adjust the center of the reflection band back to lower wavelengths, 15.0 wt% additional chiral dopant was mixed into the oligomer containing 50% Monomer 2. In this mixture, a 608 nm reflection band shift was observed upon cooling from 80 °C to 55 °C in a wavelength range similar to that of the oligomer containing no Monomer 2 (Figure 5c,d). The steepness of the curve for this mixture was 24 nm/°C, which is significantly less compared to the steepness when no additional dopant was added. However, the steepness was 4 times larger compared to the oligomer with no Monomer 2. Band shifting is usually stronger at longer wavelengths, but these results show that the steepness of the band shift is also dependent on the composition of the oligomer. It is indeed possible to obtain a steeper shifting curve in the same wavelength range by adding a smectic monomer to the monomer mixture used to prepare the oligomers.

We performed similar experiments with our new batch of oligomers, mixing 4.43 wt% chiral dopant with the oligomer with 50% Monomer 2. When the center of the reflection band was plotted as a function of the temperature, mixing the chiral dopant resulted in the appearance of the reflection band at 68 °C (Figure S6b). Above 68 °C, the reflection band showed a reversible shift between 1939 nm and 1285 nm in 12 °C. After increasing the additional amount of the chiral dopant to 10.0 wt%, the trend of the wavelength gradually dropped in the measurement range. At the same measurement temperature, the center of the reflection band blue shifted further. Using varying amounts of chiral dopant, we can adjust the position of the reflection band center and temperature-responsive range. This makes such a mixture useful for further applications such as temperature-responsive IR reflectors that can autonomously reflect infrared light and save energy when the environmental temperature changes.

## 3. Materials and Methods

### 3.1. Materials

The diacrylate liquid crystal Monomer 1 and Chiral Dopant 4 were purchased from Jiangsu Hecheng Advanced Materials Co. Ltd., Jiangsu, China. The diacrylate liquid crystal Monomer 2 was obtained from Jiangsu Creative Electronic Chemicals, Jiangsu, China. n-Butylamine 3 was received from Shanghai Macklin Biochemical Co., Ltd., Shanghai, China. Tetrahydrofuran (THF), dichloromethane (DCM), poly(vinyl alcohol) (PVA), and deuterated chloroform were all purchased from Sigma-Aldrich, China.

### 3.2. Preparation of the Cholesteric Main-Chain Oligomer

Diacrylate liquid crystal Monomer 1, diacrylate liquid crystal Monomer 2, Chiral Dopant 3, and n-butylamine were added in a vial, dissolved in THF, and stirred at room temperature (~25 °C) for 24 h. Then, the mixture was stirred at 100 °C for another 18 h, and the THF was allowed to evaporate during oligomerization. The THF was further removed in a vacuum oven at 50 °C overnight.

### 3.3. PVA-Functionalized Glass Substrate

The pre-treatment of glass substrates was carried out as reported previously. Glass substrates were cleaned by using ultra-sonication (acetone and ethanol, 30 min) and then treated in a UV–ozone photoreactor (PR-100, Ultra Violet Products, Shenzhen, China) for 20 min. The surface of the glass substrates was modified by spin-coating polyvinyl alcohol with a molecular weight of 6000 (5 wt% solution in distilled water) for 30 s at 2500 rpm. After heating for 30 min at 60 °C, the polyvinyl-alcohol-coated glass plates were cooled to room temperature and then rubbed on a velvet cloth to induce alignment.

### 3.4. Preparation of the LC Cells

The mixture containing Monomer 1, Monomer 2, Chiral Dopant 3, and n-butylamine was placed into a vacuum oven at 50 °C to make sure all the remaining solvent was removed. Then, a drop of the mixture was applied on a PVA-coated glass substrate at a workable temperature with some silicon solid particles (10 μm) onto four corners of the substrate to serve as spacers. Another slide of PVA-coated glass was placed directly on top. The glass substrate was sheared along the rubbing direction to align the mixture. The well-aligned film was then ready to test. The measurements were completed within a few days after sample preparation at most.

### 3.5. Mixing of Cholesteric Main-Chain Oligomer and Chiral Dopant

The mixture containing cholesteric main-chain oligomer and chiral dopant was dissolved in DCM and stirred at room temperature to ensure good mixing. Then, the solvent was evaporated at 40 °C and moved to a vacuum oven at 40 °C overnight to eliminate the remaining solvent. By using the previously described method, the mixture was made into a film in a cell.

### 3.6. Characterization

NMR spectra were measured on 400 MHz Varian AS400. DSC curves were recorded with a Mettler Toledo DSC 1 from Mettler Toledo. Each sample with different contents of Monomer 2 was heated from −10 °C to 120 °C, then cooled to −10 °C again. All heating and cooling rates were fixed at 5 °C/min. Thermal history was erased, and all the transition temperatures reported here were from the second heating and cooling cycle. Phase behavior was examined using a Leica CTR6000 polarized optical microscope, equipped with a Leica DFC 420C camera and Linkam PE95/T75 temperature controller. Heating and cooling rates were fixed at 1 °C/min. Phase transition information was collected from the second heating and cooling cycle. The transmittance spectra were recorded using a UV–vis–NIR spectrophotometer (Perkin Elmer Lambda 950, Perkin Elmer, Guangzhou, China). A water-agent temperature controller (EYELA NCB-1200) was used for controlling the temperature.

## 4. Conclusions

In conclusion, cholesteric main-chain liquid crystal oligomers containing various amounts of smectic monomers were successfully prepared, and their behavior was analyzed in glass cells. Steepening of the reflection band wavelength versus the temperature curve of the main-chain CLC oligomer was achieved by building increasingly larger amounts of the smectic monomer into the main-chain structure due to the increasing number of short-range smectic structures in the helical cholesteric structure. The aligned CLC film with 25% Monomer 2 showing a reversible band shift of 1479 nm from 40 °C to 75 °C was obtained. Larger reversible reflection band shift was achieved as well by increasing the concentration of Monomer 2 to 40% and 50%. More complex phase behavior was observed in POM and DSC measurements in these oligomers. Interestingly, POM observations show an increase in distance between the fingerprint structures, further indicating that the observed increase in sensitivity is caused by an enhanced pre-transitional effect. These results lead to the conclusion that the higher amount of smectic domains in main-chain oligomers greatly promote the cholesteric–smectic pre-transitional effect. Furthermore, the center of the reflection band can be easily adjusted by mixing the oligomer with an additional chiral dopant. Using this novel way, photonic reflectors with hypersensitivity can be achieved for versatile applications, where sensitive temperature response properties are required, such as smart photonic sensors, reflectors, and other potentials on smart wearable optical devices.

## Figures and Tables

**Table 1:** Different concentrations of the oligomers.

No.	Concentrations (mol%)				Monomer 2: (Monomer 1 + Monomer 2) (%)	Average DP
	Monomer 1	Monomer 2	Butylamine 3	Monomer 4		
1	47.6	0	47.4	5.0	0	2.8
2	35.8	12.0	47.2	5.0	25.0	2.1
3	28.5	19.0	47.5	5.0	40.0	1.9
4	23.8	23.8	47.4	5.0	50.0	1.8
5	19.0	28.6	47.2	5.2	60.0	1.7
6	9.6	38.2	47.1	5.1	80.0	2.1
7	0	47.6	47.3	5.1	100.0	2.8

## Data Availability

Not applicable.

## Supplementary Materials

The following supporting information can be downloaded at: https://www.mdpi.com/article/10.3390/ijms23063275/s1.
