# Supplementary material for: Controlling the Phase Behavior and Reflection of Main-Chain Cholesteric Oligomers Using a Smectic Monomer
Source: Int J Mol Sci. 2022 Mar 18;23(6):3275. doi: 10.3390/ijms23063275 (PMC8951454; doi:10.3390/ijms23063275)
Supplement: Supplementary file 1 [file ijms-23-03275-s001.zip › ijms-1571624-supplementary.pdf]

International Journal of
Molecular Sciences

MDPI

Supporting Information

1. POM Images of Pure Monomer 2

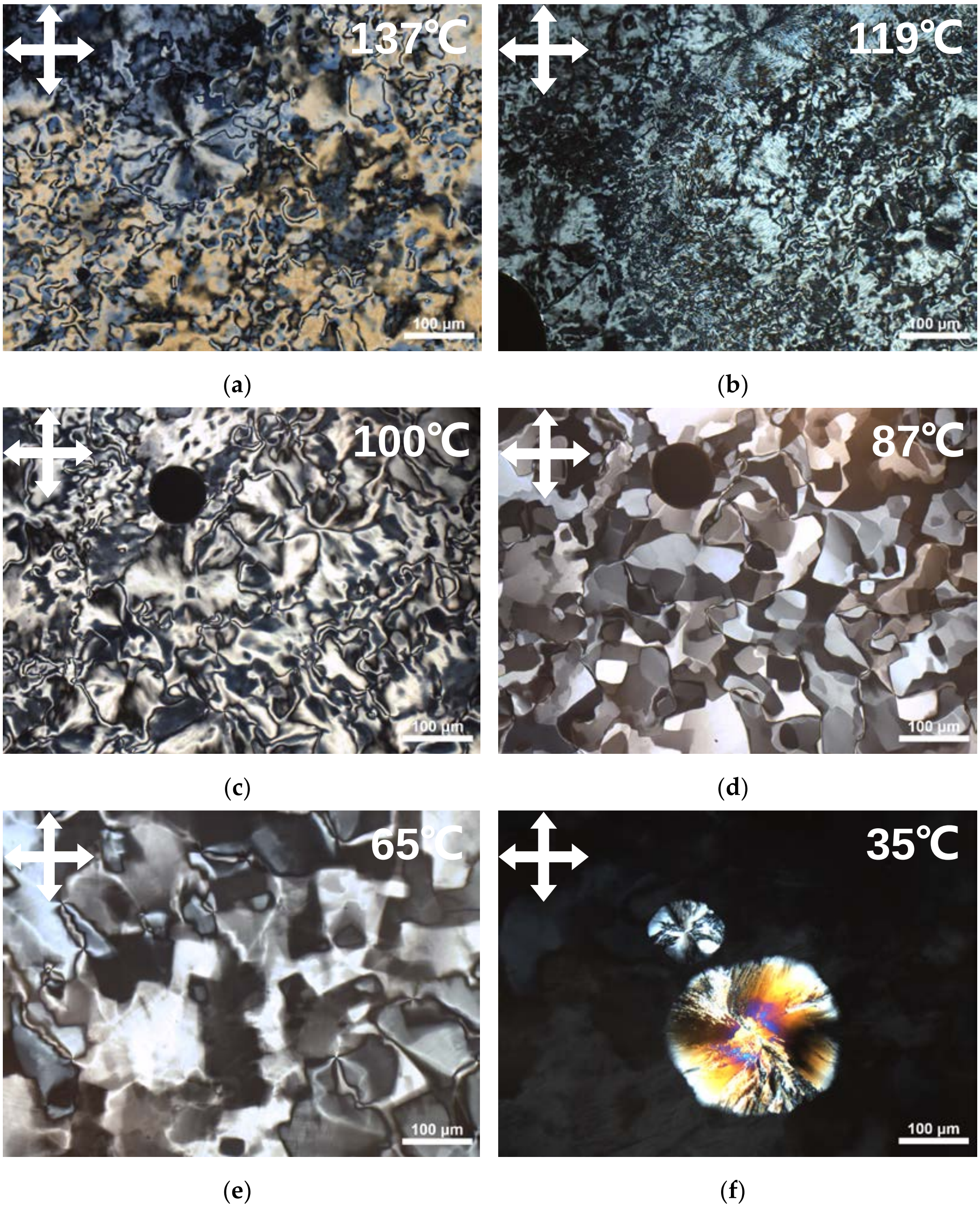

Figure S1. POM images of pure monomer 2 at different temperatures. (**a**) Textures representing a nematic phase. (**b**,**c**) extures representing a smectic phase, probably smectic A. (**d**,**e**) Textures representing a different smectic phase, probably smectic B. (**f**) Textures representing a crystalline phase.

2. ^{1}H NMR Measurement

The peak assignments used to confirm the structures and calculate the DP are marked in the spectrums. Peak **a** (2.24 ppm) corresponds to the three protons in the aromatic methyl group. Peaks **b** (5.84 ppm), **c** (6.16 ppm) and **d** (6.38 ppm) correspond to the six protons in the diacrylate end groups in the main-chain CLC oligomers. And peak **e** (8.06-8.18 ppm) corresponds to the aromatic protons in the aromatic groups, which were determined from the two different achiral diacrylate liquid crystal monomers.

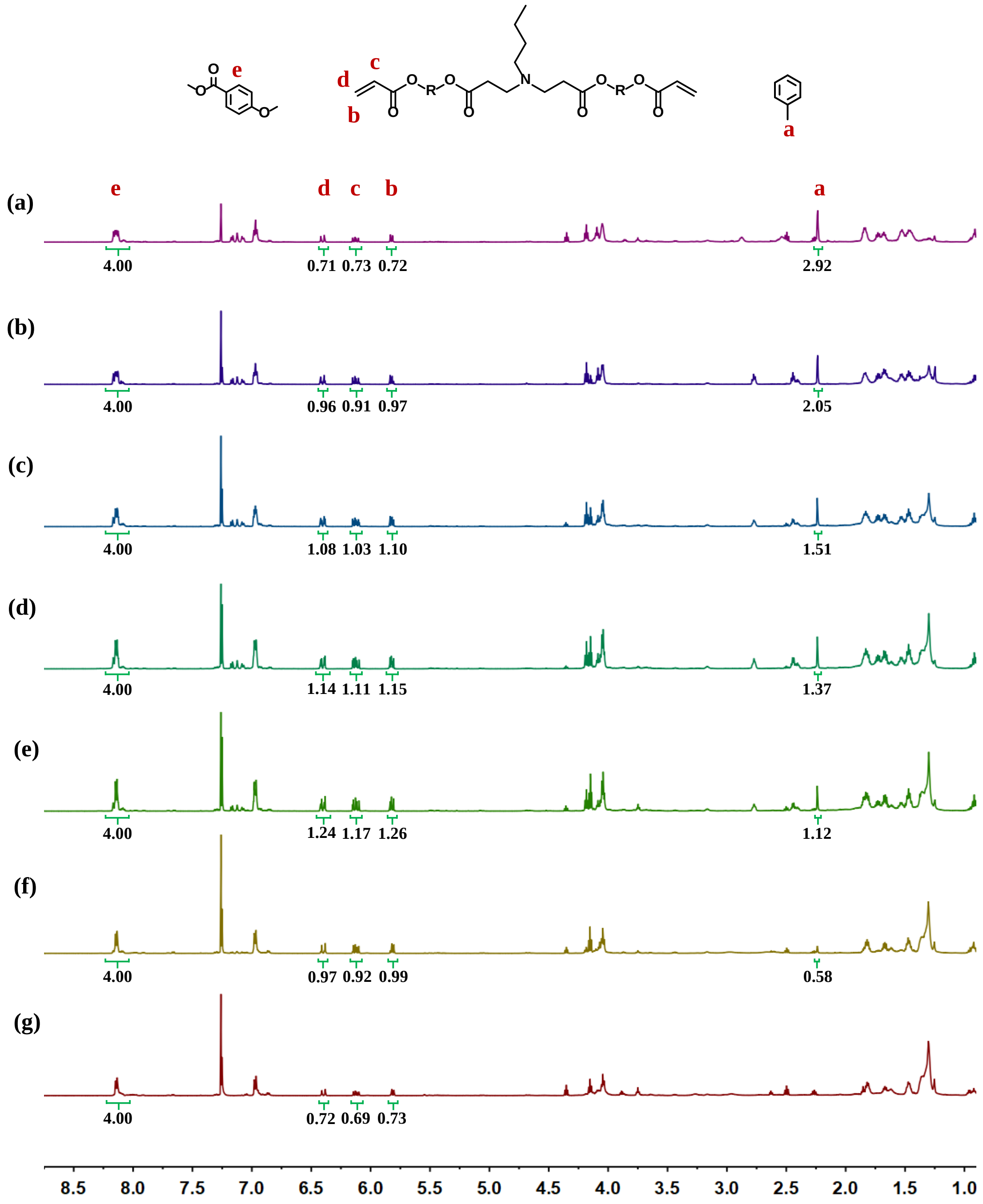

Figure S2. ^{1}H NMR spectrum of main-chain cholesteric LC oligomers with different compositions of monomer 2: (**a**) 0 %, (b) 25 %, (**c**) 40 %, (**d**) 50 %, (**e**) 60 %, (**f**) 80 %, (**g**) 100 % with peak assignments.

3. Calculation of DP from ^{1}H NMR Data

For the purpose of this study, we define the DP as the number of mesogenic units per oligomer. As the diacrylate monomer is large compared to the chain extender, we do not consider the latter as a monomer for the purpose of this definition. We also assume that the polymerization is complete and the oligomers have two acrylate end groups.

The average degree of polymerization was calculated using Eq. (1), where S_1 and S_2 are defined as the integration value of all protons in the diacrylate end groups divided by three and the integration value of the protons "e" in the mesogenic units, respectively. For each polymer chain, three sets of two protons with the same chemical environment are present in the acrylate end groups, and 4*DP protons "e" are present in its backbone. When S_2 is normalized to 4, S_1 will change proportionally, and Eq. (1) can be simplified to Eq. (2).

$$\frac{S_1}{S_2} = \frac{2}{4 \times DP} \tag{1}$$

$$DP = \frac{2}{S_1} \tag{2}$$

So, the average degree of polymerization of the oligomers were calculated as follows: DP (0 % monomer 2) = 2/0.72 ≈ 2.8, DP (25 % monomer 2) = 2/0.95 ≈ 2.1, DP (40 % monomer 2) = 2/1.07 ≈ 1.9, DP (50 % monomer 2) = 2/1.1 ≈ 1.8, DP (60 % monomer 2) = 2/1.2 ≈ 1.7, DP (80 % monomer 2) = 2/0.96 ≈ 2.1, DP (100 % monomer 2) = 2/0.71 ≈ 2.8.

4. POM Images of CLC Oligomers with Different Compositions of Monomer 2

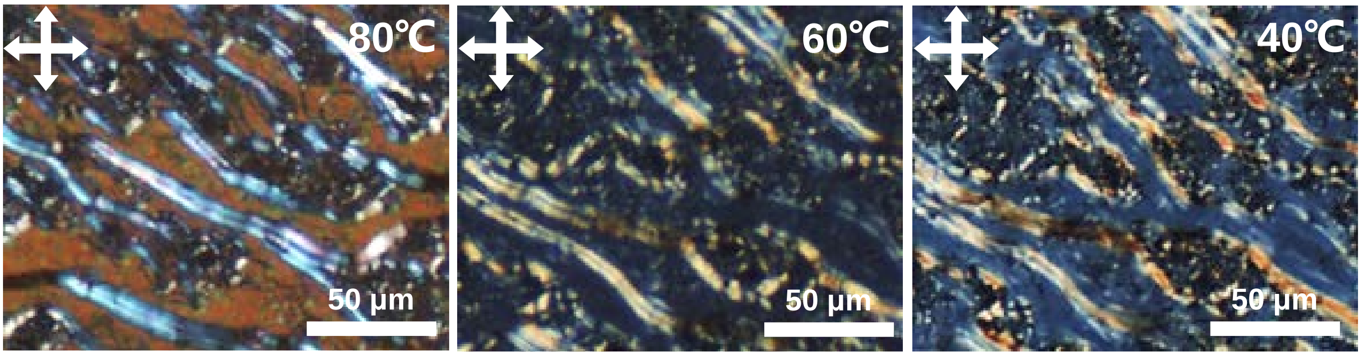

(**a**)

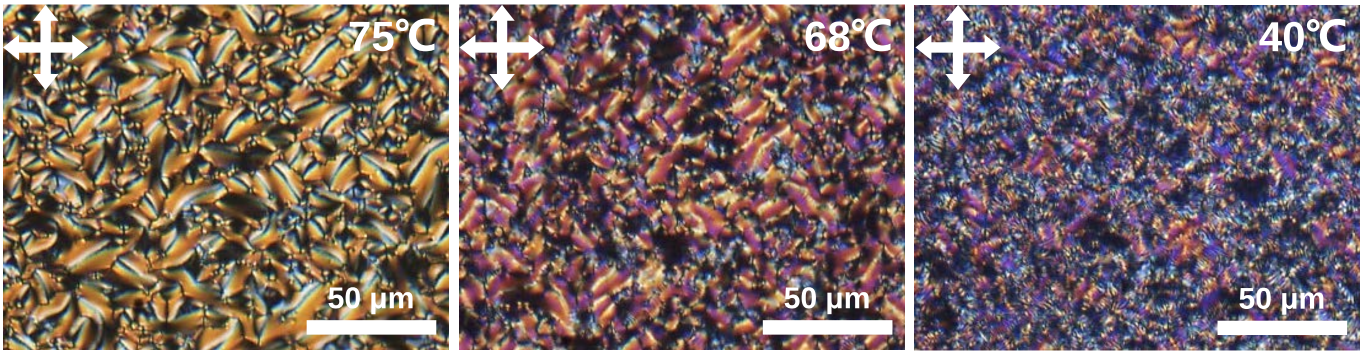

(**b**)

Figure S3. POM images of CLC oligomers with different compositions of monomer 2 under different temperatures. (**a**) 25 %, (**b**) 50 %.

5. Optical Analysis of Aligned CLC Oligomers with Different Concentrations of Monomer 2

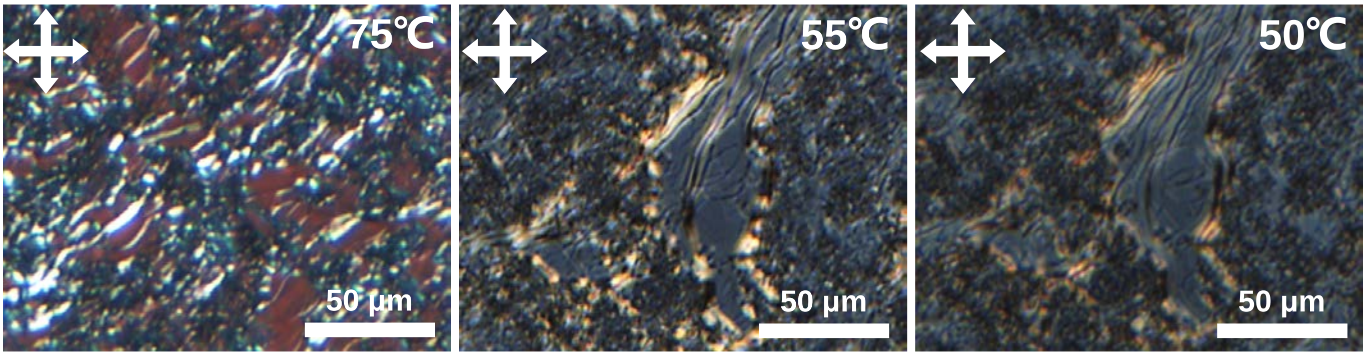

(**a**)

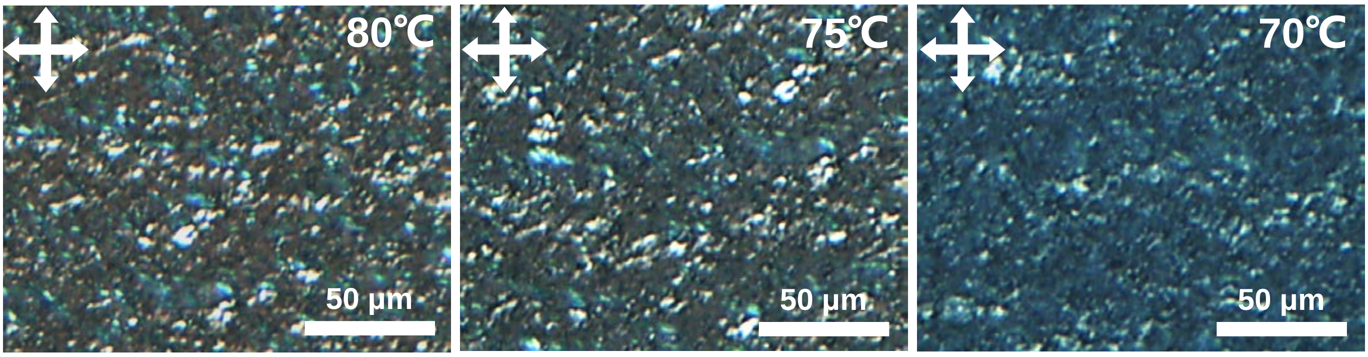

(**b**)

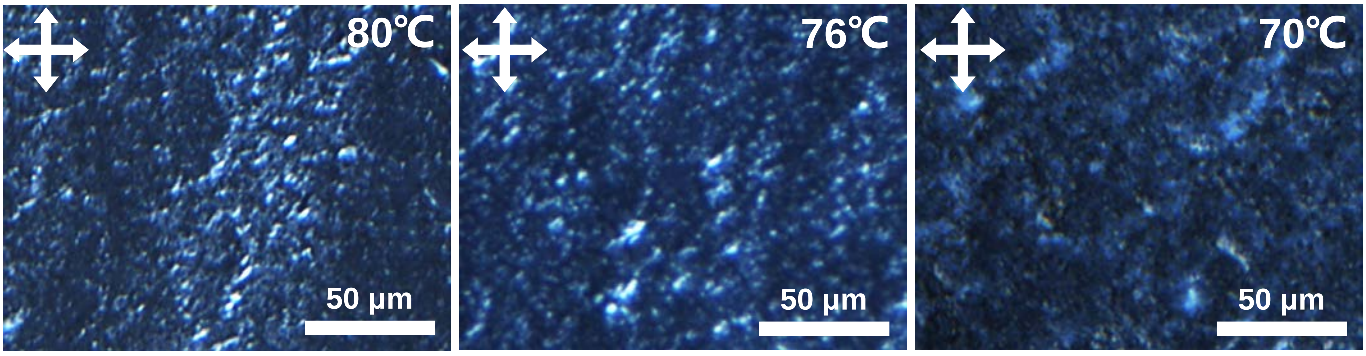

(**c**)

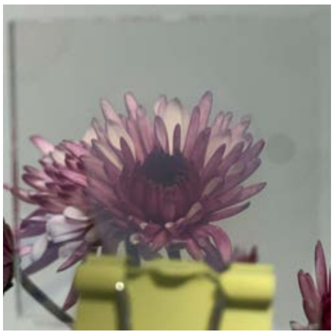

(**d**)

Figure S4. POM images of aligned CLC oligomers with (**a**) 25%, (**b**) 40%, and (**c**) 50% monomer 2 under different temperatures. (**d**) Photographic image of the aligned CLC oligomer with 25% monomer 2 in front of a flower background at a distance.

6. Temperature Response of the Reflection Band of Aligned Oligomers with 40% and 50% Monomer 2

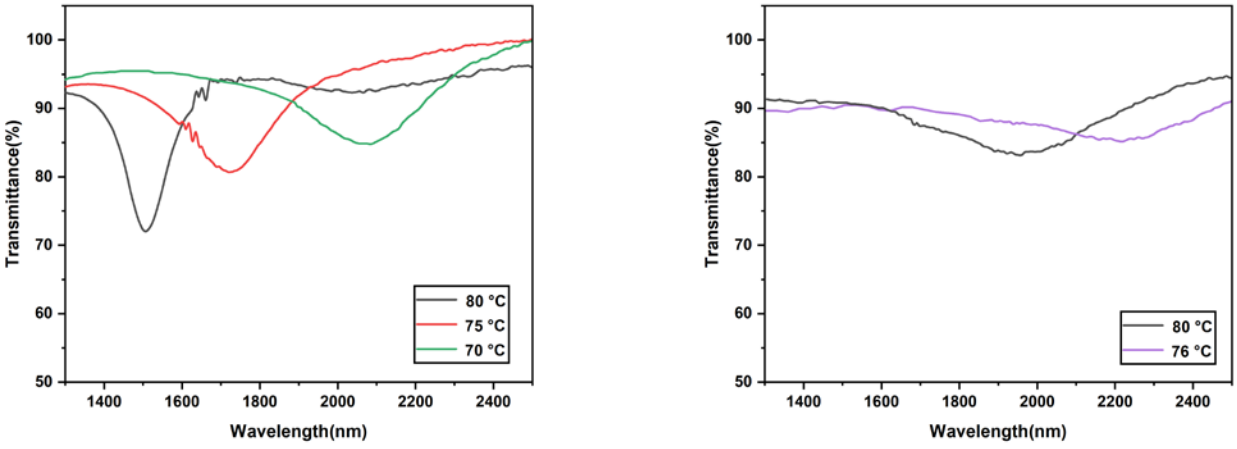

(**a**) (**b**)

Figure S5. Transmittance spectra of the aligned oligomers with (**a**) 40%, and (**b**) 50% monomer 2.

7. The Reflection Band Center of a Second Batch of Aligned Oligomers as a Function of Temperature Upon Cooling

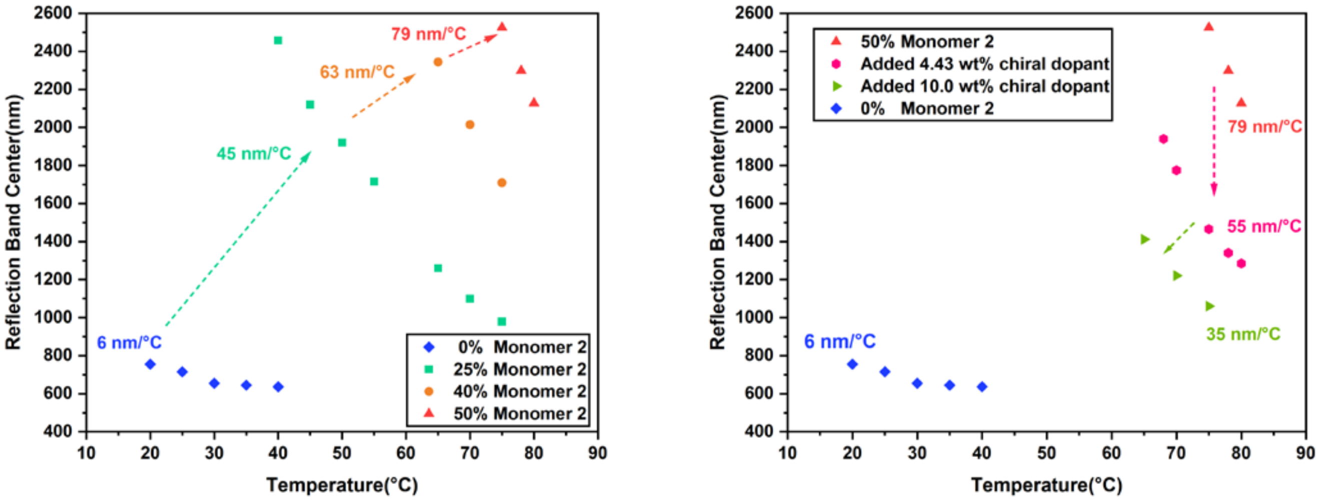

(**a**) (**b**)

Figure S6. (**a**) The central reflection wavelength of the oligomers with different concentrations of monomer 2 as a function of temperature upon cooling. (**b**) The central reflection wavelength of the oligomer containing 50 % monomer 2 after mixing with additional chiral dopant as a function of temperature upon cooling.

8. Study on the Influence of Temperature on the Helical Twisting Power

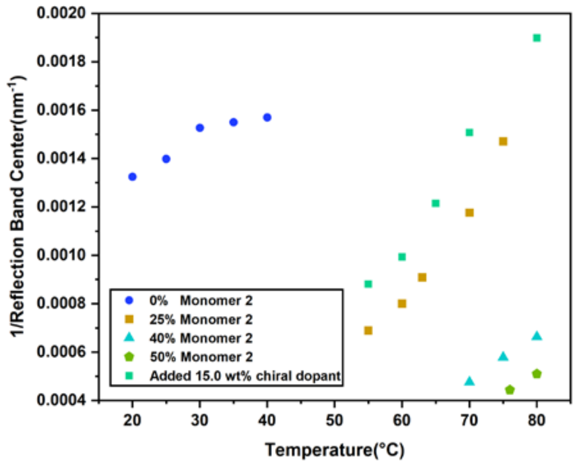

(**a**)

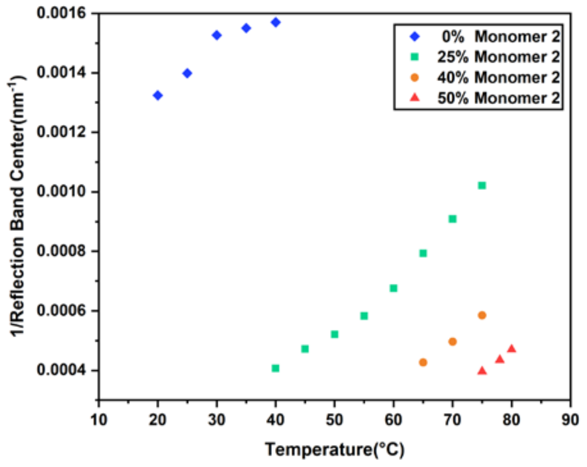

(**b**)

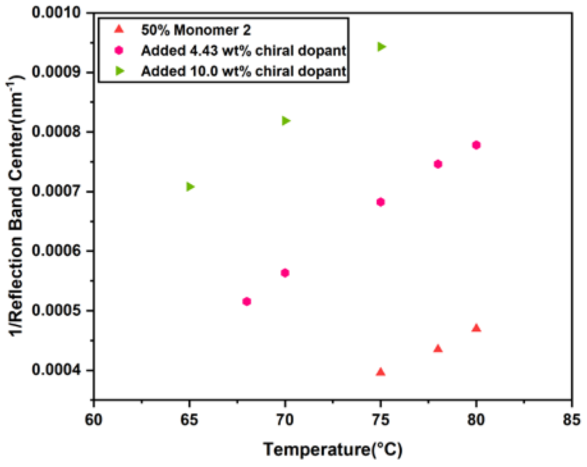

(**c**)

Figure S7. (**a**) The inverse central reflection wavelength of the oligomers with different concentrations of monomer 2 as a function of temperature. (**b**) The inverse central reflection wavelength of the new batches of oligomers with different concentrations of monomer 2 as a function of

temperature. (**c**) The inverse central reflection wavelength of the new batch of oligomers with 50 % monomer 2 after mixing with additional chiral dopant as a function of temperature.

9. Boltzmann Relation of ln(λ^{-1}) Versus T^{-1} was Studied to Search for Linearity.

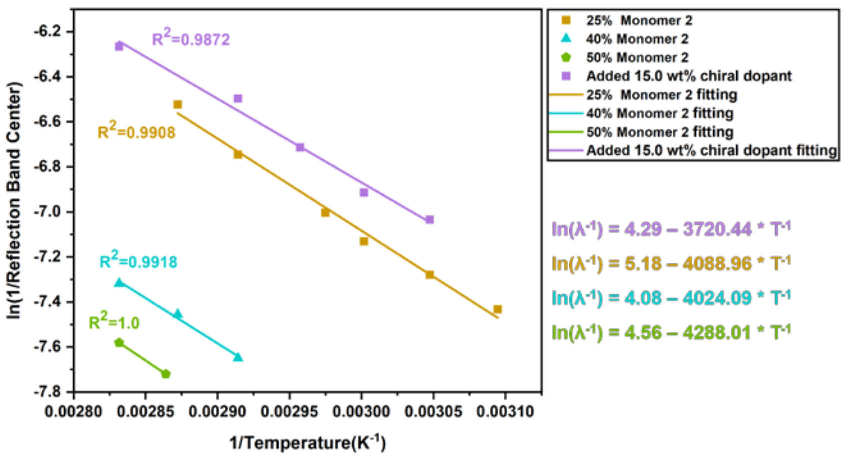

(**a**)

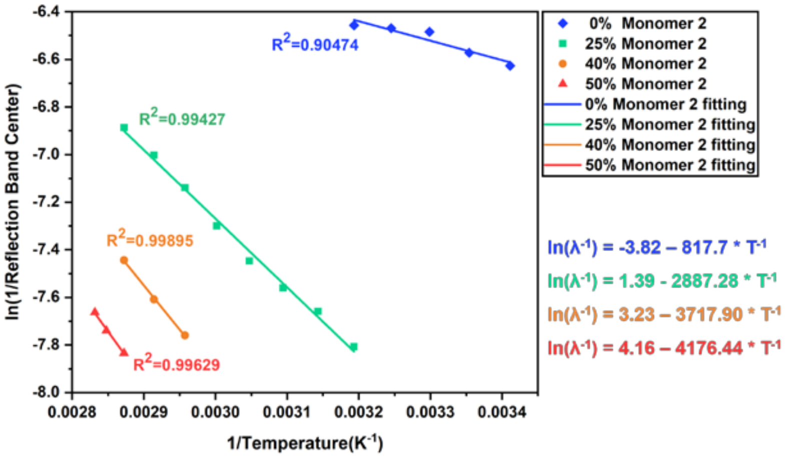

(**b**)

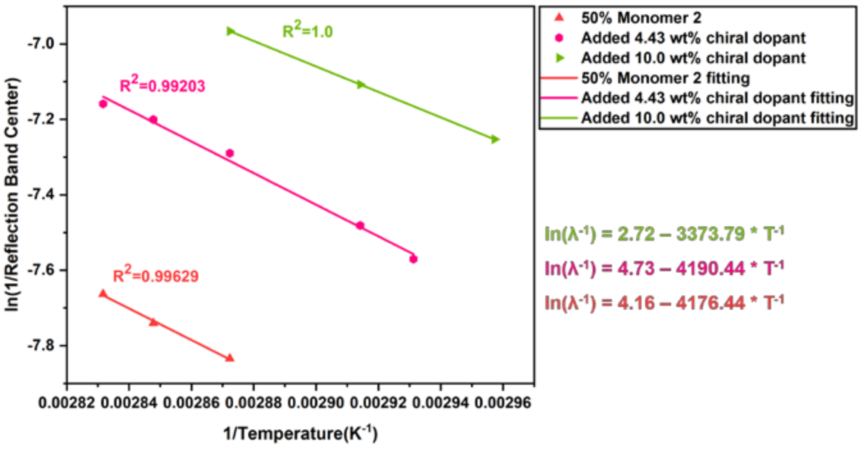

(**c**)

Figure S8. (**a**) Fitting curve of ln(λ^{-1}) versus T^{-1} of the oligomers with different concentrations of monomer 2. (**b**) Fitting curve of ln(λ^{-1}) versus T^{-1} of the new batches of oligomers with different concentrations of monomer 2. (**c**) Fitting curve of ln(λ^{-1}) versus T^{-1} of the new batch oligomers with 50 % monomer 2 after mixing with additional chiral dopant.
